# A Questionnaire-Based Survey on the Long-Term Management of Canine Leishmaniosis by Veterinary Practitioners

**DOI:** 10.3390/ani12060731

**Published:** 2022-03-14

**Authors:** Maria A. Pereira, Rute Santos, Carmen Nóbrega, Cristina Mega, Rita Cruz, Fernando Esteves, Carla Santos, Catarina Coelho, João R. Mesquita, Helena Vala, Gabriela Santos-Gomes

**Affiliations:** 1 Instituto Politécnico de Viseu, Escola Superior Agrária de Viseu, Campus Politécnico, 3504-510 Viseu, Portugal; cnobrega@esav.ipv.pt (C.N.); amega@esav.ipv.pt (C.M.); rcruz@esav.ipv.pt (R.C.); festeves@esav.ipv.pt (F.E.); casarede@esav.ipv.pt (C.S.); ccoelho@esav.ipv.pt (C.C.); hvala@esav.ipv.pt (H.V.); 2 Global Health and Tropical Medicine (GHTM), Instituto de Higiene e Medicina Tropical (IHMT), Universidade Nova de Lisboa, UNL, Rua da Junqueira 100, 1349-008 Lisboa, Portugal; santosgomes@ihmt.unl.pt; 3 CERNAS—Research Centre for Natural Resources, Environment and Society, ESAV, Instituto Politécnico de Viseu, 3500-606 Viseu, Portugal; 4 Polytechnic Institute of Portalegre, 7300-110 Portalegre, Portugal; rutesantos@ipportalegre.pt; 5 Research Centre for Endogenous Resource Valorization (VALORIZA), 7300-555 Portalegre, Portugal; 6 Centre for the Research and Technology of Agro-Environmental and Biological Sciences (CITAB), University of Trás-os-Montes e Alto Douro, 5001-801 Vila Real, Portugal; 7 Epidemiology Research Unit (EPIUnit), Instituto de Saúde Pública da Universidade do Porto, 4050-091 Porto, Portugal; jrmesquita@icbas.up.pt; 8 Laboratório para a Investigação Integrativa e Translacional em Saúde Populacional (ITR), 4050-600 Porto, Portugal; 9 ICBAS—School of Medicine and Biomedical Sciences, Porto University, 4050-313 Porto, Portugal

**Keywords:** canine leishmaniosis, questionnaire, veterinary follow-up, long-term treatment, relapse

## Abstract

**Simple Summary:**

Canine Leishmaniosis is a chronic and potentially fatal disease, caused by *Leishmania infantum*, a zoonotic microorganism. In economically disadvantaged regions, costs associated with long-term patient monitoring may determine that some owners decline veterinary follow-up of their dogs, with potentially severe implications for animal welfare and public health. This online, questionnaire-based survey aimed to assess how Portuguese veterinary practitioners perform long-term patient management and recognize relapses. More than half of the respondents stated that most dog owners declare having financial restraints, which condition the use of diagnostic tests during long-term follow-up. Allopurinol *ad aeternum* or until disease remission and domperidone were the most prescribed treatment, and relapses were detected by the reappearance or worsening of clinical signs by most veterinary practitioners. The rate of relapse detection was higher in the most economically favored regions, probably because of a lesser constraint on the use of the appropriate diagnostic tests. This study confirms that owner financial restraints negatively influence veterinary follow-up and relapse recognition, potentially compromising clinical decision making and favoring the maintenance of *Leishmania infantum* infection endemic status in Portugal.

**Abstract:**

Canine Leishmaniosis (CanL) is a chronic and potentially fatal disease. In economically disadvantaged regions, costs associated with long-term patient monitoring may determine that some owners decline veterinary follow-up of their dogs. This online, questionnaire-based survey aimed to assess how Portuguese veterinary practitioners perform long-term patient monitoring and recognize relapses. More than 50% of respondents reported that 50–100% of dog owners declared financial restraints. Hence, in these circumstances, most veterinary practitioners only performed clinical examination and serology. However, when owners did not declare financial restriction, other tests were additionally performed, such as renal and hepatic profiles, hemogram, serum protein electrophoresis and urine protein creatinine ratio. The mean number of exams performed when owners presented financial restraints was significantly lower than the number of exams performed without economic limitations. Most veterinary practitioners prescribed allopurinol *ad aeternum* or until disease remission and domperidone. CanL relapses were recognized by more than half of respondents “Always”, through the reappearance or worsening of clinical signs, whereas about a quarter detected an increase in anti-*Leishmania* antibody levels and identified abnormalities in the serum protein electrophoresis profile. The relapse rate was higher in the Lisbon Metropolitan Area and north, the most economically favored regions of Portugal. This study confirms that owner financial restraints negatively influence veterinary follow-up and relapse recognition, ultimately compromising clinical decision making and favoring the maintenance of *Leishmania infantum* infection endemicity.

## 1. Introduction

Canine leishmaniosis (CanL) is a zoonosis, mainly caused by the parasite *Leishmania infantum*, which is transmitted by blood-sucking insects. CanL is endemic in more than 70 countries in South and Central America, the Mediterranean region, Africa, and Asia [1,2]. The overall seroprevalence of *L. infantum* infection in mainland Portugal is 6.31%, ranging from 0.88% to 16.16% [3], varying from one area to another, depending on specific demographic and environmental factors that condition the habitat of the vector [1,4]. 

CanL is a chronic and potentially fatal disease. Common clinical signs include dermatological lesions, weight loss, lymphadenopathy, osteoarticular pathology, muscle atrophy, ocular lesions, and splenomegaly, among others. The most frequent clinicopathological abnormalities include anemia, thrombocytopenia, monocytosis, hyperproteinemia, and elevated blood urea and creatinine levels. Chronic kidney disease (CKD) is a common consequence of CanL progression and the main cause of death or euthanasia of affected dogs [5,6,7].

Therapy with anti-*Leishmania* drugs frequently promotes clinical cure, increases life expectancy [8,9,10] and decreases canine infectiousness to the sand fly vector [11,12,13,14]. Veterinary practitioners prescribe allopurinol plus meglumine antimoniate, allopurinol plus miltefosine or allopurinol in monotherapy, as initial anti-*Leishmania* treatment [7,15]. Long-term treatment (also known as maintenance treatment) with allopurinol and/or immuno-modulators is recommended to maintain disease remission and elicit an appropriate and more efficient immune response against the parasite [16,17]. 

Allopurinol is a purine analogue of adenosine nucleotides that blocks the synthesis of RNA by the parasite, and acts as a leishmaniostatic drug [18], decreasing the number of parasites without eliminating them [19]. However, allopurinol treatment can lead to increased urinary xanthine levels, due to the inhibition of the enzyme xanthine oxidase [20], ultimately producing urolithiasis and renal mineralization [20,21,22]. Furthermore, drug resistance has been reported in *L. infantum* parasites, isolated from relapsed dogs treated with allopurinol [23], which raises concern about its long-term administration.

Domperidone is an antagonist of the dopamine D2 receptor that reversibly increases prolactin blood levels. This drug proved therapeutic efficacy by reducing clinical signs and *Leishmania* antibody levels, while improving cellular immune *Leishmania*-specific response [16]. Active hexose correlated compound (AHCC) and nucleotides, which present immuno-modulatory properties, have been proposed as an alternative treatment to allopurinol, especially for dogs that manifest allopurinol-related adverse effects [17]. 

Despite adequate treatment, dogs rarely achieve a parasitological cure, and relapses are frequent after treatment ends, or even during allopurinol long-term therapy [24,25]. A relapse occurs when a patient returns to manifest clinical signs (clinical relapse) and/or laboratory abnormalities (clinicopathological relapse) of CanL, after a temporary improvement (remission) [26]. Nevertheless, CanL complete remission might not be achieved, hampering the identification of relapses. On the other hand, in endemic regions of zoonotic visceral leishmaniasis, if adequate preventive measures are not implemented, there is a possibility of reinfection, which can be mistaken for relapse. 

CanL relapses have been associated with a marked increase in *L. infantum* antibody levels and parasite loads, which can enhance the infectivity of dogs for sand fly vectors [5,27,28,29]. Early detection of relapses requires periodic and long-term patient monitoring. Clinical monitoring consists of ongoing patient assessment throughout the course of the disease [6], and includes clinical examination, assessment of the clinicopathological parameters and inflammatory status of the patient, evaluation of *Leishmania*-specific immune response, and quantification of parasite load, in tissues where *L. infantum* typically establishes a latent infection (bone marrow, spleen, lymph nodes) [30]. However, the costs associated with patient monitoring, particularly in economically disadvantaged regions, may determine that some owners decline the veterinary follow-up of their dogs, which may have severe implications for animal welfare and public health. Nevertheless, the zoonotic character of *L. infantum* infection requires a One Health approach, which includes early diagnoses and adequate control of CanL, maintaining disease remission in affected dogs [7,31,32]. 

Although, LeishVet [5,33], Canine Leishmaniasis Working Group (CLWG) [6,34] and the International Renal Interest Society (IRIS) [35] have provided evidence-based consensus on the clinical management of CanL and treatment of *L. infantum*-associated CKD, the ideal frequency of monitoring, laboratory tests performed during veterinary follow-ups, long-term therapeutic protocol [30,36,37] and the best way to identify and manage CanL relapses have not yet been adequately defined, which complicates decision making and the work of veterinary practitioners. 

Albeit several questionnaire-based surveys have been conducted in Europe since the last decade, focusing on diagnoses, treatment protocols, prevention strategies, and application of the current guidelines by veterinary practitioners on CanL management [15,38,39,40,41,42,43,44,45,46], none have addressed the long-term management of this disease.

Thus, this questionnaire-based survey aimed to assess how Portuguese veterinary practitioners perform long-term management of CanL, including long-term treatment, nature and frequency of laboratory exams implemented in follow-ups, and clinical and clinicopathological findings that allow recognition of relapses, while highlighting the possible consequences of owner financial status on clinical decisions. 

## 2. Materials and Methods

### 2.1. Survey Development and Distribution

A questionnaire was developed using an online platform (Google Forms^®^). The questionnaire was prepared in Portuguese language and consisted of 21 questions. The 21 questions were closed-ended or checkboxes, but in five of them, by filling in the option “Other” respondents could give an answer that was not present among the listed options. The questionnaire covered four main topics, specifically: characterization of veterinary practitioners and practices (7 questions), diagnoses and veterinary follow-up (8 questions), long-term treatment (2 questions), and relapses (4 questions) (Appendix A Table A1—questionnaire with English translation).

Veterinary practitioners were asked to consider dogs suspected of having CanL, those presenting clinical signs (clinical suspicions) and/or clinicopathological abnormalities (laboratory suspicions) compatible with CanL, and CanL diagnoses in those dogs with compatible clinical signs and/or clinicopathological abnormalities in which the infection was confirmed by serological tests, Polymerase Chain Reaction (PCR) or direct diagnoses (parasite identification in cytological preparation from skin, lymph node, bone marrow etc.). Veterinary practitioners were asked to consider dogs that did not respond to treatment, those that did not improve clinically and/or clinicopathologically in the first three months of treatment, and relapse when a patient returned to manifesting clinical signs (clinical relapse) and/or laboratory abnormalities (clinicopathological relapse) of CanL after a temporary improvement. These descriptions were included in the questionnaire.

For internal validation, the questionnaire was evaluated by the authors and by a group of veterinary practitioners. The minimum number of responses to the questionnaire calculated considering a population size of 6667 (number of active members in the Portuguese College of Veterinarians, OMV) with a confidence level of 95% and a margin of error of 8%, was 147. Sample size was estimated using an online calculator (Calculator.net). The questionnaire was diffused via a Portuguese social network in a veterinary group with 4300 members and sent twice by email, four weeks apart to 885 practices in mainland Portugal.

The questionnaire was anonymous, and the respondents were informed of and agreed to the collection of data for scientific purposes before answering the survey. Furthermore, the questionnaire was approved by the ethics committee of the Instituto Politécnico de Viseu, Viseu, Portugal.

### 2.2. Data Processing and Statistical Analysis

All data were collected using Google Forms^®^ and downloaded in a database (Microsoft Excel 2016^®^; Microsoft Corp., Redmond, WA, USA). To operationalize numerical variables, the range of values that appear in the answer options were converted to the mean value of the range, for example, 1–5 was converted to 3, 6–10 to 8, >30 to 35, etc. [44]. The ratio between the number of relapses per year and the number of diagnoses per year was calculated to obtain an approximate yearly incidence of relapse. The 18 districts of mainland Portugal were grouped under the Nomenclature of Territorial Units for Statistics (NUTS) II regions with minor adaptations.

Statistical analysis was performed with SPSS v.27.0 (IBM Corp., Armonk, NY, USA, 2020). Descriptive statistics were used to analyze the categorical data. Data exhibited non-normal distribution in all variables (Shapiro–Wilks test), and therefore non-parametric tests were used in the statistical analysis of numerical variables. Specifically, Kruskal–Wallis test with paired comparison was used to compare CanL diagnoses and relapse rate among different NUTS II regions; Kendall test with pairwise comparison was used to compare the number of CanL diagnoses, suspects and follow-ups; and Mann–Whitney test to compare the number of exams carried out with and without financial restraints. Statistical significance of non-parametric tests was set at *p* < 0.05. 

## 3. Results

### 3.1. Characterization of Veterinary Practitioners and Practices

**Figure 1:**
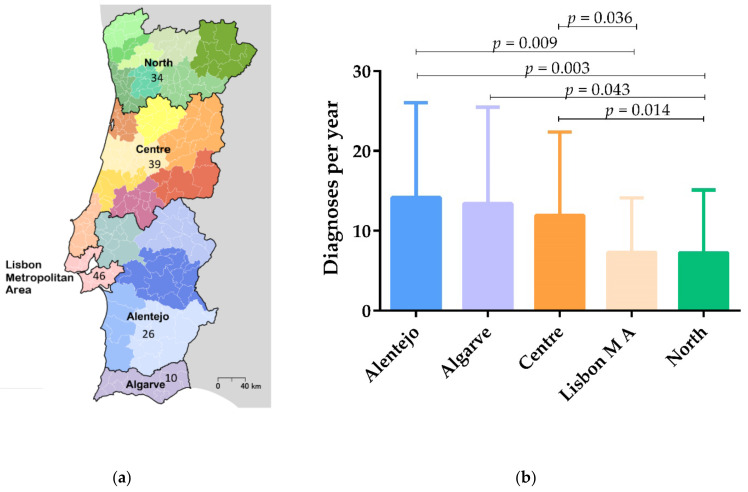
Distribution of respondents and canine leishmaniosis (CanL) diagnoses by NUTS II regions. (**a**) Number of veterinary practitioners who responded to the questionnaire by NUTS II regions. (**b**) CanL diagnoses among different NUTS II regions, represented by means and standard deviations. Statistical analysis was performed using the non-parametric Kruskal–Wallis test with paired comparison (*p* < 0.05).

One hundred and fifty-five replies were obtained from veterinary practitioners working in the 18 geographical districts of mainland Portugal, grouped in five NUTS II regions (Figure 1a). Most of the veterinary practitioners worked at Veterinary Health Care Centers (VHCC) (96.1%), 2.6% at Official Companion Animal Shelter Centers, one (0.6%) at an Association of Animal Protection and another (0.6%) refereed a different type of practice. The practices were in urban (54.8%), peri-urban (22.6%) or rural (22.6%) areas. Most of the practices were of small dimensions, with just 1–2 (48.4%) or 3–4 (28.4%) veterinary practitioners, and only 23.2% of the practices employed five or more veterinary practitioners. 

Most veterinary practitioners worked exclusively in small animal practices (83.2%), 14.8% had a mixed activity (small and large animals) and 2% have chosen the option “Other”. Respondents had been working as veterinary practitioners for 13.4 ± 8.7 years (mean ± SD). Most practices had a low workload, with 7.7% of the respondents examining <5 animals, and 61.9% 5–10 animals per day. Only 30.3% of the respondents examined >10 animals per day.

### 3.2. Canine Leishmaniosis Suspected Cases, Diagnoses, and Veterinary Follow-Ups

The percentage of veterinary practitioners that estimated 1–5, 6–10, 11–15, 16–20, 21–30 or >30 CanL suspects per year was 17.4%, 25.8%, 16.8%, 11.6%, 7.1% and 21.3%, respectively. The percentage of veterinary practitioners that estimated 1–5, 6–10, 11–15, 16–20, 21–30 or >30 CanL diagnoses per year was 47.7%, 20%, 9%, 8.4%, 5.8%, and 8.4%, respectively, with one respondent (0.6%) from Oporto district reporting no confirmed CanL cases. When these ranges of values were converted in the mean value of the range, the number of dogs suspected of having CanL and diagnosed with the disease per year was 10.0 ± 9.7 and 8.0 ± 7.8, respectively.

The number of CanL diagnoses per year was statistically different (*p* = 0.006) among different NUTS II regions of mainland Portugal. Specifically, the number of CanL diagnoses was significantly lower in the North when compared with the Centre (*p* = 0.014), Algarve (*p* = 0.043) and Alentejo (*p* = 0.003), as well as in the Lisbon Metropolitan Area when compared with Centre (*p* = 0.036) and Alentejo regions (*p* = 0.009). The number of CanL diagnoses was slightly higher in the Alentejo region when compared with the Algarve region, but without statistical significance (Figure 1b).

Most veterinary practitioners scheduled follow-ups every 6 months (56.1%) and 27.7% every 4 months. Only 7.7% of the respondents performed follow-ups once a year and 8.4% chose “Other” frequencies. Regarding the number of veterinary follow-ups, 55.5% of the veterinary practitioners only followed 1–5 patients with CanL, 18.7% about 6–10, 12.3% about 11–15, 0.65% about 16–21, 9% about 21–30, and 3.2% >30 patients per year. Two respondents (1.3%), one from the Oporto district and another from the Lisbon district, reported that they did not follow cases of CanL.

Veterinary practitioners were asked to indicate the percentage of dogs (<25%, 25–50%, 50–75% and 75–100%) they followed-up with economic restraints. About 23.2% of veterinary practitioners reported that <25% of dog owners declared financial restraints, 24.5% reported financial restrictions in 25–50%, 31.6% in 50–75% and 20.6% in 75–100% of CanL cases. 

**Figure 2:**
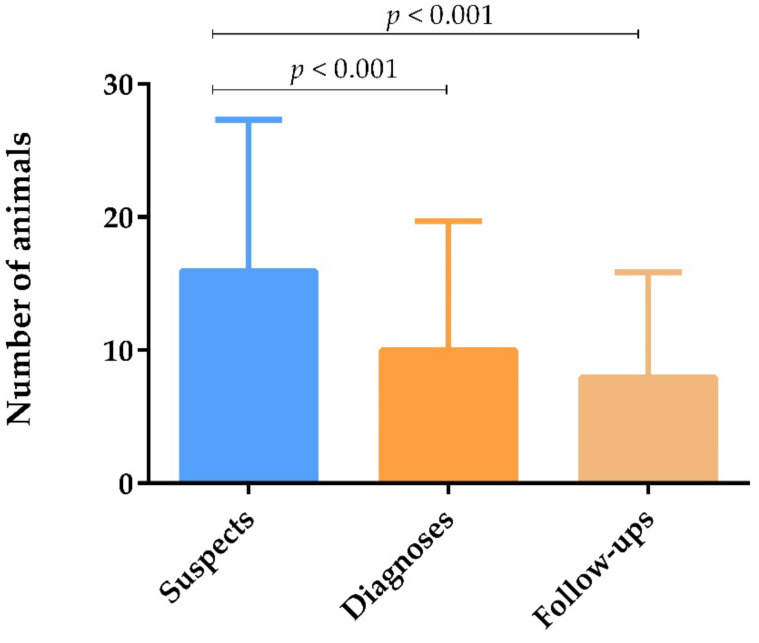
Number of dogs suspected of having CanL, diagnosed with CanL, and subjected to veterinary follow-up yearly, represented by means and standard deviations. Statistical analysis was performed using the non-parametric Kendall test with pairwise comparison (*p* < 0.05).

Comparing the number of CanL suspected cases, diagnoses, and veterinary follow-ups, it was observed that the number of dogs suspected of having CanL was significantly higher than the number of dogs diagnosed with the disease and followed-up (*p* < 0.001) (Figure 2). Respondents attributed the low percentage of veterinary follow-ups mainly to financial restraints (32.9%), lack of awareness and compliance (31.1%), and negligence by dog owners (9.7%).

### 3.3. Long-Term Veterinary Follow-Up without Financial Restraints

**Figure 3:**
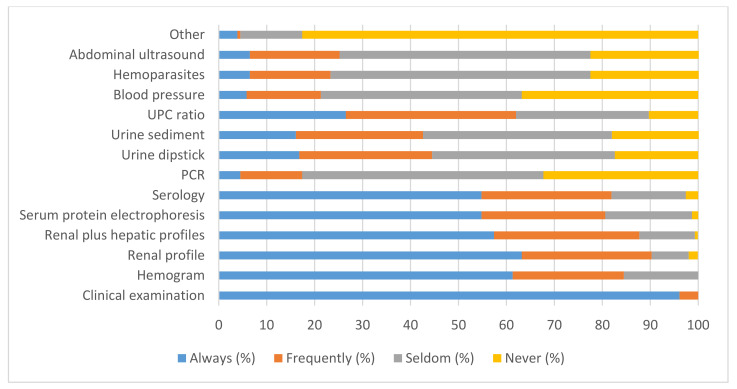
Exams performed by veterinary practitioners during the follow-up of dogs with CanL when owners did not refer to financial restraints.

When owners did not refer to financial restraints, most veterinary practitioners state that they “Always” or “Frequently” performed the clinical examination, renal profile, renal plus hepatic profiles, hemogram, quantification of anti-*Leishmania* antibodies, serum protein electrophoresis profile and Urine Protein Creatinine (UPC) ratio during follow-ups. Most veterinary practitioners “Seldom” or “Never” carried out PCR test, blood pressure measurements, specific tests to diagnose hemoparasite infections, abdominal ultrasound, urine sediment analysis and urine dipstick. Some respondents performed “Other” tests, such as cytology (1.9%), X-ray (1.3%), and determination of C-reactive protein (CRP) (0.6%) (Figure 3). 

### 3.4. Long-Term Veterinary Follow-Up with Financial Restraints

When owners declared financial restraints, most veterinary practitioners stated that they prioritized (choose “Always” or “Frequently”) the clinical examination (79.4%) and serology (55.5%). The complete biochemical profile (renal plus hepatic) (49.0%), serum protein electrophoresis (46.5%), renal profile (44.5%), hemogram (41.9%), and UPC ratio (23.2%) were selected by less than half of the respondents. Less than 10% of veterinary practitioners performed urinary sediment analysis (9.7%), urine dipstick (6.5%), PCR (6.5%), blood pressure measurements (5.2%), specific tests to diagnose hemoparasite infections (3.9%), abdominal ultrasound (1.9%) and “Other” exams (1.9%).

**Figure 4:**
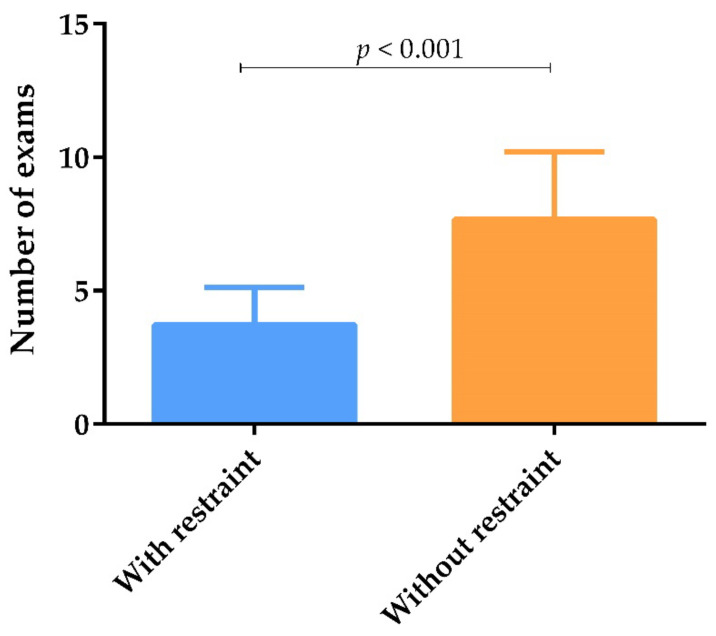
Number of exams carried out by veterinary practitioners according to owner financial status, represented by means and standard deviation. The mean number of exams was calculated by adding the exams carried out “Always” and “Frequently” by veterinary practitioners. Statistical analysis was performed using the Mann–Whitney test (*p* < 0.05).

The mean number of clinicopathological exams performed by veterinary practitioners in each dog during the veterinary follow-up with financial restraints (3.9) was significantly lower than the number of exams performed without financial restraint (7.7) (*p* < 0.001) (Figure 4). 

### 3.5. Long-Term Treatment of Canine Leishmaniosis

**Figure 5:**
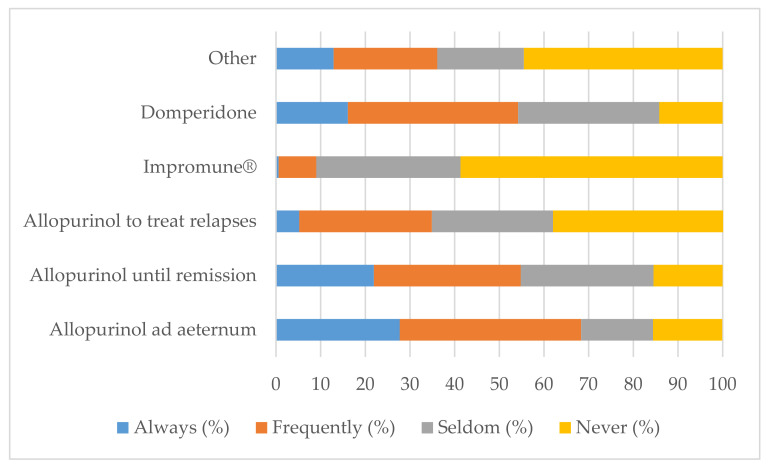
Different long-term treatments prescribed by veterinary practitioners to treat CanL.

Almost 70% of veterinary practitioners stated that they “Always” or “Frequently” prescribe allopurinol ad aeternum as maintenance treatment for CanL. Just over 50% “Always” or “Frequently” selected allopurinol until disease remission and domperidone, and a minor percentage selected allopurinol to treat relapses. Less than 10% “Always” or “Frequently” elected nucleotides (Impromune^®^). Veterinary practitioners that choose the option “Other”, reported the administration of the leishmanicidal drugs, miltefosin (27.7%) and meglumine antimoniate (25.2%); food supplements containing *Coriolus versicolor* (3.9%); corticosteroids (1.3%); benazepril (1.3%); therapeutic diets (1.3%); vitamins (1.3%); antibiotics (0.6%); acupuncture (0.6%); hemoacupuncture (0.6%); aminosidin (0.6%); nutraceuticals, such as fat acids and antioxidants (4.5%) (Figure 5). 

### 3.6. Identification of Relapses by Veterinary Practitioners

Veterinary practitioners estimated that 1.8 ± 1.9 dogs per year did not improve clinically and/or clinicopathologically in the first three months of treatment, which corresponded to 34% of the dogs followed-up yearly. The estimated number of animals that relapsed was 2.6 ± 2.4, which relates to 43% of the dogs followed-up yearly.

**Figure 6:**
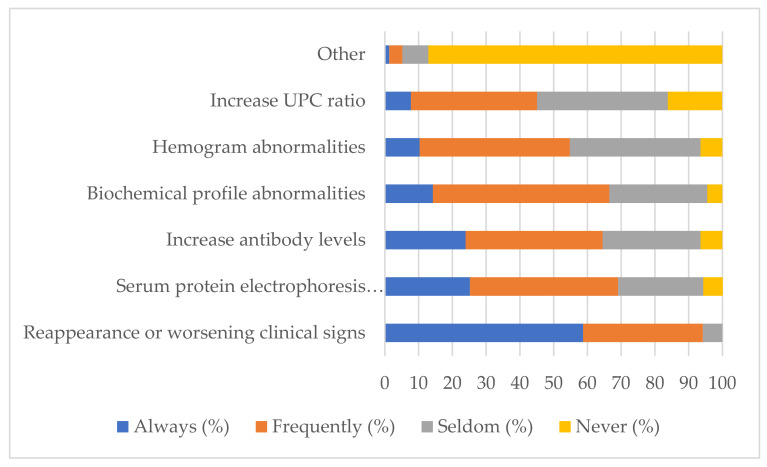
Different clinicopathological exams employed by veterinary practitioners to identify relapses of CanL.

Most veterinary practitioners stated that they recognized relapses “Always” through the reappearance or worsening of the clinical signs. About a quarter of veterinary practitioners recognized CanL relapses “Always” through the identification of abnormalities in the serum protein electrophoresis profile and increased levels of anti-*Leishmania* antibodies. Abnormalities in the biochemical profile, hemogram and UPC ratio were employed by a small percentage of veterinary practitioners. Real-time PCR was employed by a very small percentage (0.6%) of veterinary practitioners (Figure 6).

According to 33% of the respondents, 50–100% of the relapses occurred between 1–3 years after initial diagnoses, and only 27% of the veterinary practitioners recognized 50–100% of the relapses >5 years after initial CanL diagnoses (Table 1).

**Figure 7:**
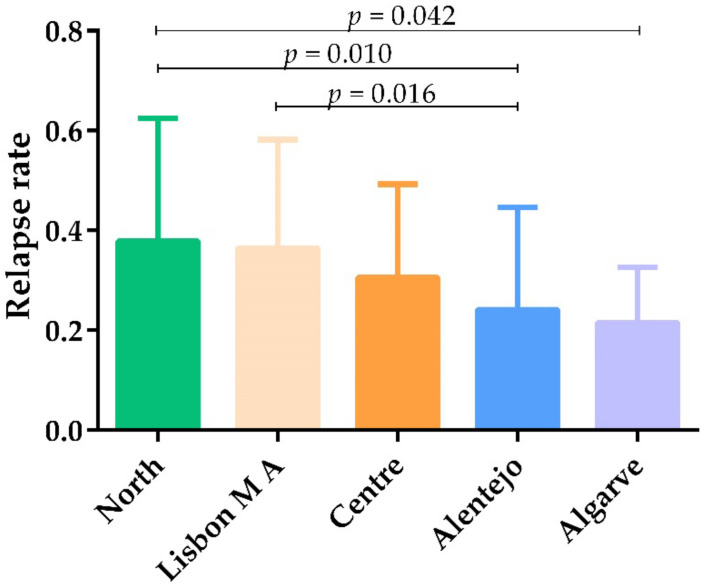
Relapse rate at different NUTS II regions of mainland Portugal. Statistical analysis was performed using the non-parametric Kruskal–Wallis test with pairwise comparison (*p* < 0.05).

The relapse rate was statistically different (*p* = 0.031) among the NUTS II regions. In the North region, the relapse rate was significantly higher than in the Algarve region (*p* = 0.042). On the contrary, the relapse rate in Alentejo was significantly lower than in the Lisbon Metropolitan Area (*p* = 0.016) and North (*p* = 0.010) regions. The relapse rate was slightly higher in the North region, when compared with the Lisbon Metropolitan Area, but without statistical significance (Figure 7). 

## 4. Discussion

Veterinary questionnaire surveys represent a quick and cost-effective alternative method to assess the current status of CanL and its clinical management, allowing one to obtain data from large geographic areas [38,47].

This survey collected information about long-term follow-up, long-term treatment, and identification of relapses of dogs with leishmaniosis, from 155 veterinary practitioners in mainland Portugal. Despite the effort to publicize the questionnaire, the number of veterinary practitioners who answered was somewhat low. However, the number of replies was similar to other recent questionnaire surveys carried out in Portugal [15,39,42]. Furthermore, this survey included responses from veterinary practitioners working in all districts of mainland Portugal, which allow us to compare data from veterinary practitioners working in different regions. Although it is necessary to interpret the data obtained in this survey with caution, since it results from an opinion poll, being subject to potential sources of bias and the risk of under- or over-reporting [38], these results may serve as a basis for further studies on this overlooked subject.

According to our data, the number of CanL diagnoses were higher in Algarve, Centre and Alentejo regions. Confirming our results, the last national survey identified higher seroprevalences in Beja and Portalegre districts, in the Alentejo region and in Castelo Branco, in the Centre region, as new endemic areas [3], and in another study, high seroprevalences were identified in the Algarve region [48]. 

Careful veterinary follow-up of dogs with CanL, after initial treatment, and throughout the animal’s life, is essential for the identification of relapses [6] and may improve the prognosis [27]. However, in the present study, the number of dogs undergoing veterinary monitoring was significantly lower than the number of CanL diagnoses per year, pointing out to the refusal of veterinary follow-up by some owners, which may have severe One Health implications. Respondents attributed these low rates of veterinary follow-up to financial restraints, lack of awareness and compliance, or negligence by dog owners. 

As the clinical presentation of CanL can be extremely variable, it is not possible to define a common and standardized laboratory procedure to be used during veterinary follow-up. However, it is widely accepted that during the follow-up, clinical examination should be performed together with hematology and biochemical profile, serum protein electrophoresis and urinalysis, including the assessment of UPC ratio, paying particular attention to previously altered parameters [6,30]. As recommended, most Portuguese veterinary practitioners “Always” or “Frequently” performed the clinical examination, together with hematology and biochemical profile, serology, serum protein electrophoresis and UPC ratio during CanL follow-ups. 

However, it should be noted that just over a quarter of respondents “Always” performed the UPC ratio and, less frequently, the urine dipstick analysis. Nevertheless, the early identification of proteinuria is essential, since this abnormality is a marker of kidney disease and a risk factor for the progression of CKD [30,49]. The urine dipstick colorimetric test is the usual first-line screening test for detection of proteinuria, since it is inexpensive and easy to use, although sensitivity and specificity are relatively low, whereas the UPC ratio allows for the confirmation and quantification of proteinuria [35]. According to LeishVet guidelines, UPC ratio should be included in the follow-up of proteinuric dogs [5,33]; equally, the American College of Veterinary Internal Medicine (ACVIM) recommend assessing proteinuria in dogs with a disease that potentially induces CKD [50], as is the case with CanL. 

The prevalence of systemic arterial hypertension in dogs with CanL is 29% [51] and higher (61.5%) in dogs with glomerular disease, secondary to CanL. Hypertension may even be present in the earlier stages of CanL before azotemia becomes apparent [52]. Since hypertension is associated with proteinuria and progression of renal damage, dogs with CanL and CKD must be screened for the presence of hypertension [35,51]. However, the percentage of Portuguese veterinary practitioners that “Always” or “Frequently” performed arterial pressure measurements was less than 22%.

The present study clearly shows that patient follow-up is negatively affected by the financial restraints of dog owners. When dog owners declare financial restrictions, most veterinary practitioners prioritized the clinical examination and serology, avoiding other exams that are essential to identify clinicopathological abnormalities, namely, early stages of CKD, characterized by few extra-renal clinical signs [35]. Thus, the ability to identify clinicopathological relapses is compromised, since it depends on the ability to carry out the appropriate laboratory exams for a complete patient evaluation. 

Long-term treatment of dogs with CanL can reduce relapses [53], delay the progression of renal damage [54] and decrease the infectivity of dogs for the vector [13]. However, there are few studies comparing the efficacy of the different therapeutic options for maintenance treatment of CanL and, when available, the period under investigation was limited [17,26].

Allopurinol is the most widely studied drug for the long-term treatment of CanL and, probably for this reason, is the most frequently prescribed by Portuguese veterinary practitioners. However, several administration protocols are described, namely, the administration for 6–12 months [5,33], for longer periods, or even *ad aeternum* [25,27], and just for one week to a month [55]. The diversity of protocols for allopurinol administration, employed by Southern European veterinary practitioners, has been previously documented [42]. In this survey, most veterinary practitioners “Always” or “Frequently” prescribed allopurinol *ad aeternum* or until CanL remission, and one-third of the respondents prescribed allopurinol, specifically, to treat CanL relapses. To clarify, the best protocol for allopurinol administration that sustains remission, avoiding resistance, is essential to develop further clinical trials.

Since allopurinol can lead to the formation of xanthine crystals and uroliths along the urinary tract, it is strongly recommended to include the urine sediment analysis and abdominal ultrasound in the laboratory workup of dogs under long-term treatment with allopurinol protocols [30]. However, our results reveal that less than 50% of the veterinary practitioners performed urine sediment analysis and less than 30% abdominal ultrasound “Always” or “Frequently” during CanL follow-ups, being unable to identify the side effects associated with allopurinol administration. 

More than half of the Portuguese respondents “Always” or “Frequently” prescribed the immuno-modulator, domperidone, which contrasts with the low frequency (11%) of its therapeutic use by Spanish veterinary practitioners [43]. On the contrary, Impromune^®^ (Bioibérica, Spain), a food supplement containing nucleotides and AHCC with immunomodulatory properties was “Always” or “Frequently” administered by less than 10% of respondents, despite having been attributed an efficacy similar to allopurinol, and absent of side effects [17,56]. Even without strong scientific evidence, some respondents employed nutraceuticals, including vitamins, essential fatty acids, antioxidants, and Corpet^®^ (Mycology Research Laboratories Ltd., Bedfordshire, UK), a mushroom (*Coriolus versicolor*) nutrition product, for the long-term treatment of CanL. 

Veterinary practitioners stated that one-third of dogs did not improve clinically and/or clinicopathologically in the first three months of treatment. The absence of therapeutic response can be attributed to an incorrect diagnosis, concomitant diseases, or drug resistance. Since clinical signs and clinicopathological abnormalities of CanL are variable and nonspecific; the management of a dog suspected of having leishmaniosis should include differential diagnosis to rule out other concomitant infectious or non-infectious diseases that mimic CanL signs [5,33,57,58]. Co-infections with other pathogens are frequently reported in dogs with leishmaniosis, affecting the severity of the disease and worsening the prognosis [7,59,60,61,62,63,64]. Furthermore, it is important to reinforce that low levels of anti-*Leishmania* antibodies are not a good indicator of CanL, and further work-up is necessary to confirm or exclude the disease [5,33].

Due to the relative inefficacy of CanL treatment in promoting parasite clearance, relapses are frequent. Most veterinary practitioners that answered the questionnaire recognized relapses by noticing the reappearance or worsening of the clinical signs (clinical relapse). Serum protein electrophoresis abnormalities and the increase in anti-*Leishmania* antibodies were also frequently used as criteria to identify relapses. Serum protein electrophoresis profile is considered a good monitoring tool and its sequential analysis informs of the evolution of the inflammatory status of the patient. Abnormalities in the serum protein profile are correlated with the severity of clinical scores [30,65]. 

Regarding quantitative serology, despite being frequently used as a monitoring tool, its usefulness is controversial, since antibody levels were related to the clinical evolution of CanL by some authors [66,67], but not by others [25,68]. Quantitative PCR provides information about parasite burden, which may be useful for patient follow-up. As occurs with quantitative serology, it is advisable to obtain baseline values at the time of diagnosis for further comparison during the follow-up [30]. However, Portuguese veterinary practitioners rarely employ PCR as a monitoring tool, probably due to the high costs of the test. However, the absence of evidence-based consensus on the best way to identify and manage CanL relapses can hamper the effort of veterinary practitioners. 

According to Portuguese veterinary practitioners, more than 40% of dogs followed-up yearly relapse, mainly, between the first and third year after CanL diagnoses, but relapses can occur at any time point, as previously described [8,25]. Interestingly, relapse recognition (relapse rate) was higher in the Lisbon Metropolitan Area and Northern region, the wealthiest regions of Portugal, probably due to greater purchasing power of dog owners living in these regions, which allow for better veterinary follow-up.

This study confirms that owners’ financial restraints negatively influence veterinary follow-up and relapse recognition in dogs with CanL, ultimately compromising clinical decision making and favoring the maintenance of *Leishmania infantum* infection endemicity.

## 5. Conclusions

Overall, this survey provides a global picture of the long-term management of CanL, based on data provided by local veterinary practitioners. The results of this study support the general perception that the economic resources of populations negatively influence the veterinary care provided to pets. Indeed, the financial restraints of owners living in economically disadvantaged regions negatively influence veterinary follow-up and relapse recognition of CanL, ultimately compromising clinical decision making, animal welfare and public health. Furthermore, the variability of therapeutic and follow-up protocols used by veterinary practitioners suggest a need for standardized guidelines, indicating the best long-term treatment options and follow-up protocols for this disease, concerning, mainly, CanL relapses.

## Figures and Tables

**Table 1:** Percentage of veterinary practitioners that estimated relapses at different time points along the course of CanL. The values 0–25%, 25–50%, 50–75% and 75–100% represent percentage of dogs that relapsed.

	0–25%	25–50%	50–75%	75–100%
3–12 months	45.2	36.8	14.8	3.2
1–3 years	27.1	40.0	29.0	3.9
3–5 years	46.5	29.0	20.0	4.5
>5 years	51.6	21.3	16.1	11.0

## Appendix A

**Table A1:** Questionnaire used to assess how Portuguese veterinary practitioners perform long-term management of CanL and recognize relapses.

Topics	Questions	Options Frequency	Type of Question
Characterization of veterinary practitioners and practices	How many years have you been working as a veterinary practitioner?		Short answer
	Where do you work?	Veterinary Health Care Center/Official Companion Animal Collection Center/Association of Animal Protection/Other	Multiple option
	How many veterinary practitioners work at your practice?	1/2/3–5/5 or more	Multiple option
	Estimate how many animals you examine per day. The answer should only reflect YOUR clinical practice.	<5/5–10/>10	Multiple option
	How do you define your current clinical practice?	Pets only/mixed clinic (small and large animals)/other	Multiple option
	How do you define the location of your practice?	Rural/urban/sub-urban	Multiple option
	In which district do you practice?		Multiple option
Diagnoses and veterinary follow-up	Estimate the number of animals you identify as suspects of canine leishmaniasis per year.	0/1–5/6–10/11–15/16–20/21–30/>30	Multiple option
	Estimate the number of dogs you diagnose with canine leishmaniosis annually.	0/1–5/6–10/11–15/16–20/21–30/>30	Multiple option
	Estimate how many dogs you follow-up with leishmaniasis annually.	0/1–5/6–10/11–15/16–20/21–30/>30	Multiple option
	Estimate the percentage of dogs with leishmaniasis that you follow-up with financial restraints.	<25%/25–50%/50–75%/75–100%	Multiple option
	How often do you use each of the following maintenance treatment in dogs with leishmaniasis?	Allopurinol *ad aeternum*/allopurinol until disease remission/allopurinol to treat relapses/domperidone/Impromune^®^/Other Always/Frequently/Seldom/Never	Multiple option
	If you use other treatment, specify which one or which ones.		Short answer
	How often do you recommend follow-ups for animals with canine leishmaniasis?	Every 4 months/every 6 months/Once a year/Other frequency	Multiple option
	If using other frequency, specify.		Short answer
	How often do you use each of these tests in the follow-up of dogs with canine leishmaniasis without financial restraints?	Clinical examination/hemogram/renal profile/renal plus hepatic profile/serum protein electrophoresis/serology/PCR/urine dipstick/urine sediment/urine protein creatinine ratio/blood pressure measurements/specific tests to diagnose hemoparasite infections/abdominal ultrasound/other Always/Frequently/Seldom/Never	Multiple option
	If using “Other” exam, specify.		Short answer
	When there is financial restraint, which test(s) do you prioritize for monitoring dogs with leishmaniasis?	Clinical examination/hemogram/renal profile/renal plus hepatic profile/serum protein electrophoresis/serology/PCR/urine dipstick/urine sediment/urine protein creatinine ratio/blood pressure measurements/specific tests to diagnose hemoparasite infections/abdominal ultrasound/other	Multiple option Can choose more than one option
	If using other exam, specify.		Short answer
Long-term treatment	Do you think that all animals with canine leishmaniasis are monitored by a veterinary practitioner?	Yes/No/Don’t know	Multiple option
	If you answered “No” to the previous question, please indicate the cause.		Short answer
Relapses	Estimate how many animals do not respond to the initial anti-*Leishmania* treatment annually.	0/1/2/3/4/5/6/7–10/10	Multiple option
	Estimate in how many animals do you identify a relapse annually.	0/1/2/3/4/5/6/7–10/10	Multiple option
	How do you identify a relapse of canine leishmaniasis?	Increase in the levels of anti-*Leishmania* antibodies/reappearance or worsening of the clinical signs/identification of abnormalities in the serum protein electrophoresis profile/abnormalities in the UPC ratio/abnormalities in the hemogram/abnormalities in the biochemical profile	Multiple option
	If identified with other exams, specify.		Short answer
	In your experience, when do relapses occur?	Between 3–12 months after diagnoses/1–3 years after diagnoses/3–5 years after diagnoses/>5 years after diagnoses25%/25–50%/50–75%/75–100%	Multiple option
